# mRNA Booster Vaccination Enhances Antibody Responses against SARS-CoV2 Omicron Variant in Individuals Primed with mRNA or Inactivated Virus Vaccines

**DOI:** 10.3390/vaccines10071057

**Published:** 2022-06-30

**Authors:** Biyan Zhang, Jianxin Huo, Yuhan Huang, Shuan Yong Teo, Kaibo Duan, Yanfeng Li, Lim Kai Toh, Kong Peng Lam, Shengli Xu

**Affiliations:** 1 Singapore Immunology Network, Agency for Science, Technology and Research, 8A Biomedical Grove, Singapore 138648, Singapore; zhang_biyan@immunol.a-star.edu.sg (B.Z.); huo_jianxin@immunol.a-star.edu.sg (J.H.); huang_yuhan@immunol.a-star.edu.sg (Y.H.); teo_shuan_yong@immunol.a-star.edu.sg (S.Y.T.); duan_kaibo@immunol.a-star.edu.sg (K.D.); 2 Genscript, 164 Kallang Way, East Wing, #06-12, Singapore 349248, Singapore; yanfeng.li@genscript.com; 3 Doctors for Life Medical, 03 Pickering Street, #01-02, Nankin Row, Singapore 048660, Singapore; limkai.toh@gmail.com; 4 Department of Microbiology and Immunology, Yong Loo Lin School of Medicine, National University of Singapore, 5 Science Drive 2, Singapore 117545, Singapore; 5 School of Biological Sciences, College of Science, Nanyang Technological University, 60 Nanyang Drive, Singapore 637551, Singapore; 6 Department of Physiology, Yong Loo Lin School of Medicine, 2 Medical Drive, MD9, National University of Singapore, Singapore 1175493, Singapore

**Keywords:** vaccines, SARS-CoV-2, omicron, booster, antibodies, B cells, COVID-19

## Abstract

The advent of the Omicron variant globally has hastened the requirement for a booster vaccination dose to confer continuous protection against symptomatic SARS-CoV2 infection. However, different vaccines are available in different countries, and individuals who had adverse reactions to certain vaccine types require heterologous vaccine boosters. To understand the efficacy of different vaccination regimens in inducing humoral responses to SARS-CoV2, we examined plasma antibodies and frequencies of Omicron RBD-specific B cells in individuals who had different priming-booster vaccination regimens. We found that individuals with three homologous doses of mRNA vaccines had higher levels of IgG of all subclasses against RBD of Omicron than individuals with three homologous doses of inactivated virus vaccine. A booster with mRNA vaccine resulted in significant increases in median levels of RBD-reactive IgG1 (17–19 fold) and IgG3 (2.3–3.3 fold) as compared to individuals receiving inactivated virus booster shots regardless of priming vaccine types. More importantly, individuals who received a booster dose of mRNA vaccine, irrespective of the priming vaccine, had antibodies with higher neutralizing capability against the Omicron variant than those who received a booster dose of inactivated virus vaccine. Corroborating the antibody results, boosting with the mRNA vaccine increased the frequencies of Omicron RBD-binding B cells by (1.5–3.3 fold) regardless of priming vaccine types. Together, our data demonstrate that an mRNA vaccine (BNT162b2 or mRNA-1273) booster enhances humoral responses against the Omicron variant in individuals vaccinated with either two prior doses of mRNA or inactivated virus vaccine (CoronaVac or BBIBP-CorV), potentially providing more effective protection against SARS-CoV-2 infection, particularly by the Omicron variant.

## 1. Introduction

The ongoing global fight against SARS-CoV-2 has been made more effective with the rapid development and introduction of different vaccines. However, the arms race against the rapid emergence of new variants of concern (VOC) has prompted a constant re-evaluation of vaccine effectiveness against new strains with increased transmissibility and/or virulence. In addition, with evidence of waning protection from the vaccines over time, there is an increasing urgency in many countries to introduce booster shots to continue maintaining herd immunity [1,2,3,4]. In Singapore, three vaccines have been included under the National Vaccine Programme (NVP), including mRNA vaccines BNT162b2 by Pfizer-BioNTech/COMIRNATY, mRNA-1273 by Moderna, and inactivated virus vaccine CoronaVac by Sinovac [5]. Other approved vaccines not in the NVP include inactivated virus vaccine BBIBP-CorV by Sinopharm, and adenovirus-vector vaccines ChAdOxnCoV-19 and Ad26COVS1 by AstraZeneca and Janssen, respectively [5]. The NVP began in January 2021, with around 95% of the eligible population receiving the complete vaccination regimen by March 2022 [5]. In addition, booster shots were introduced in July 2021 based on emerging scientific data about waning vaccine protection over time and the efficacy of vaccines against new VOC.

The advent of the Omicron variant (Pango lineage B.1.1.529) has raised concerns globally as it contains 37 amino acid substitutions in its spike protein, with 15 of them in the RBD, suggesting a high potential for immune evasion with current vaccines, which target the original Wuhan-Hu-1 strain [6,7]. The Omicron variant was reported to have increased transmissibility, incidences of reinfection, and reduced neutralization by antibodies elicited by two-dose regimens of multiple types of vaccines, including mRNA, adenoviral vector, protein, and inactivated virus-based vaccines, and even in convalescent patients [6,8,9,10,11]. Therefore, it is imperative for us to evaluate the requirement and effectiveness of various booster regimens against this new VOC. In addition, the severe allergic reaction to mRNA vaccines reported in some individuals prompted the need to find an effective heterologous vaccination booster strategy to maintain protection against SARS-CoV2 in this group of vaccine recipients [12,13]. Being one of the few countries with different types of vaccines included in the NVP, Singapore is uniquely positioned to study the effectiveness of homologous versus heterologous vaccine booster regimens against SARS-CoV2 VOC within a single population [5].

Here, we examined four different groups of volunteers receiving (1) two doses of mRNA vaccines plus an mRNA vaccine booster (homologous mRNA vaccine booster); (2) two doses of inactivated virus vaccines plus an inactivated virus vaccine booster (homologous inactivated virus vaccine booster); (3) two doses of mRNA vaccines plus an inactivated virus vaccine booster (heterologous inactivated virus vaccine booster); and (4) two doses of inactivated virus vaccines plus an mRNA vaccine booster (heterologous mRNA vaccine booster). We evaluated the levels of antibodies against the RBD of the Omicron variant, the neutralizing capability against the Omicron variant compared to the ancestral strain (Wuhan-Hu-1) and Delta variant, and lastly, the frequencies of Omicron RBD-binding B cells in these four groups of volunteers. These results are important for evaluating the relative effectiveness of each booster regimen against Omicron, thereby providing information to enact better public health policies nationwide and globally.

## 2. Methods

### 2.1. Study Design, Sample Size, and Participants

Prior approval had been granted by institutional review boards (IRBs) under the Agency for Science Technology and Research (A*STAR) to Singapore Immunology Network for the conduct of this study. Healthy donors who were above the age of 21 and due for their COVID-19 vaccine booster were recruited under a Singapore Immunology Network study entitled, “Study of B cell immune responses to SARS-CoV2 vaccine”. These donors either received the mRNA (BNT162b2, Pfizer-BioNTech/COMIRNATY or mRNA-1273, Moderna) or an inactivated virus (CoronaVac, Sinovac or BBIBP-CorV, Sinopharm) vaccine as their third dose booster. These individuals were not infected with SARS-CoV2 before or during the study. Peripheral blood was collected before the booster dose and on day 28 (±5) after the booster. A total of 66 subjects were recruited for this study, 29 males and 37 females. Written informed consent was obtained from all donors in accordance with the Declaration of Helsinki for Human Research.

### 2.2. Plasma and Peripheral Blood Mononuclear Cell (PBMCs) Isolation and Storage

Whole blood was collected from our healthy donors in K3 EDTA Vacuette tubes (Greiner #455036) via venipuncture and processed within one hour of collection based on density centrifugation at 400 g for 30 min at room temperature (RT) using Ficoll–Paque Plus solution (Cytiva #17-1440-03). Undiluted plasma was collected and stored at −80 °C. After lysing red blood cells, PBMCs were frozen in freezing media (Neat fetal bovine serum with 10% Dimethyl sulfoxide), stored at −80 °C, and subsequently stored in liquid nitrogen before analyses.

### 2.3. Enzyme-Linked Immunosorbent Assay (ELISA)

Plasma samples were analyzed by ELISA to detect Omicron RBD-specific antibodies of various isotypes, including IgM, IgG1, IgG2, IgG3, and IgG4; 96-well Maxisorp plates (Thermo Fisher Scientific, Waltham, MA, USA) were coated with recombinant Omicron RBD protein (Acro Biosystem, SPD-C522e) at 2μg/mL overnight. Plates were blocked with 2% Bovine serum albumin in PBS at RT for 1 h before the addition of serially diluted plasma samples and incubated for 2 h at RT. RBD-specific antibodies of various isotypes were detected with Horseradish peroxidase (HRP)–conjugated mouse anti-human IgM (SouthernBiotech #2023-05), IgG1 (#9054-05), IgG2 (#9060-05), IgG3 (#9210-05), or IgG4 (#9200-05) antibodies. Plates were developed with stabilized chromogen tetramethylbenzidine (TMB) (Invitrogen) and stopped with 3N hydrochloric acid before reading at 450 nm on a microplate reader (Tecan). Quantification of each antibody isotype was performed using a 6-point standard curve generated from a reference control plasma sample.

### 2.4. GenScript cPass Neutralization Antibody Detection Kit

Plasma samples were analyzed by the cPass Neutralization Antibody Detection kit (Genscript), according to the manufacturer’s instructions [14]. Briefly, the plasma samples were diluted (1:10) before mixing with HRP-conjugated RBD (Ancestral strain Wuhan-Hu-1, Delta B.1.617.2 or Omicron B.1.1.529 variant) at a volume ratio of 1:1 and incubated at 37 °C for 30 min. Next, the mixture was added to wells in the capture plate and incubated at 37 °C for another 15 min. After washing, TMB solution was added to the wells, and the plate was incubated at 25 °C for 15 min. Absorbance at 450 nm was read immediately with a microplate reader (Tecan) after adding the stop solution. The percentage of inhibition was calculated following the manufacturer’s instructions.

### 2.5. Antigen-Specific B Cell Staining and Flow Cytometry Analysis

Before staining with antibodies and RBD tetramers, B cells were enriched from PBMCs using a Pan B cell Isolation Kit (Miltenyi Biotech, #130-101-638) and LS column (#130-042-401); 1 × 10^6^ B cells were incubated with BD Horizon™ Fixable Viability Stain 510 for 15 min at room temperature in the dark. After washing, the cells were stained at 4 °C, protected from light for 30 min, using a panel of fluorochrome-conjugated primary antibodies and RBD tetramers in the optimized concentrations. The antibodies in the panel, mouse anti-human CD3 (SK7), CD56 (NCAM16.2), CD14 (M5E2), CD19 (HIB19), CD20 (2H7), CD27 (M-T271), IgD (IA6-2), CD38 (HB-7, BioLegend), CD10 (HI10a, BioLegend), CD21 (B-ly4, BD Biosciences), CD38 (HIT2), CD138 (MI15), IgM (UCH-B1), and IgG (G18-145), were purchased from BD Biosciences. Fluorescent-conjugated Omicron RBD-APC and -PE proteins were prepared by mixing biotinylated RBD protein (Acro Biosystem, SPD-C82E4) and Streptavidin-APC or -PE in a 4:1 molar ratio and incubated for 15 min at room temperature. Flow cytometry was performed on a BD LSRII (BD Biosciences), and data were acquired with BD FACSDiva Software (8.0.2) and analyzed using FlowJo software (version 10).

### 2.6. Statistical Analysis

Statistical analysis was done using GraphPad Prism version 9.0.0 (GraphPad Software). Unpaired comparisons between two groups were made using Mann–Whitney U tests, while comparisons between multiple groups were made using Kruskal–Wallis tests followed by Dunn’s post hoc analyses to correct for multiple comparisons. For paired analysis between samples from different time points from the same donors, Wilcoxon matched-pairs signed-rank tests were performed. *p* values less than 0.05 are considered significant.

## 3. Results

### 3.1. COVID-19 Prime-Booster Regimens and Cohort Characteristics

A total of 66 healthy participants were divided into four groups, namely volunteers who received (1) two doses of mRNA vaccines plus an mRNA vaccine booster (homologous mRNA vaccine booster) [*n* = 22]; (2) two doses of inactivated virus vaccines plus an inactivated virus vaccine booster (homologous inactivated virus vaccine booster) [*n* = 21]; (3) two doses of mRNA vaccines plus inactivated virus vaccine booster (heterologous inactivated virus vaccine booster) [*n* = 13]; and (4) two doses of inactivated virus vaccines plus an mRNA vaccine booster (heterologous mRNA vaccine booster) [*n* = 10]. The two priming doses of vaccines in each volunteer were taken 21 (±7) days apart, according to the Singapore NVP Guidelines. Cohort demographics and vaccination status are summarized in Data Table 1.

### 3.2. Homologous or Heterologous mRNA Booster Dose Induces Higher Levels of Plasma IgG1 and IgG3 against RBD of Omicron Variant Than Booster with Inactivated Virus

**Figure 1:**
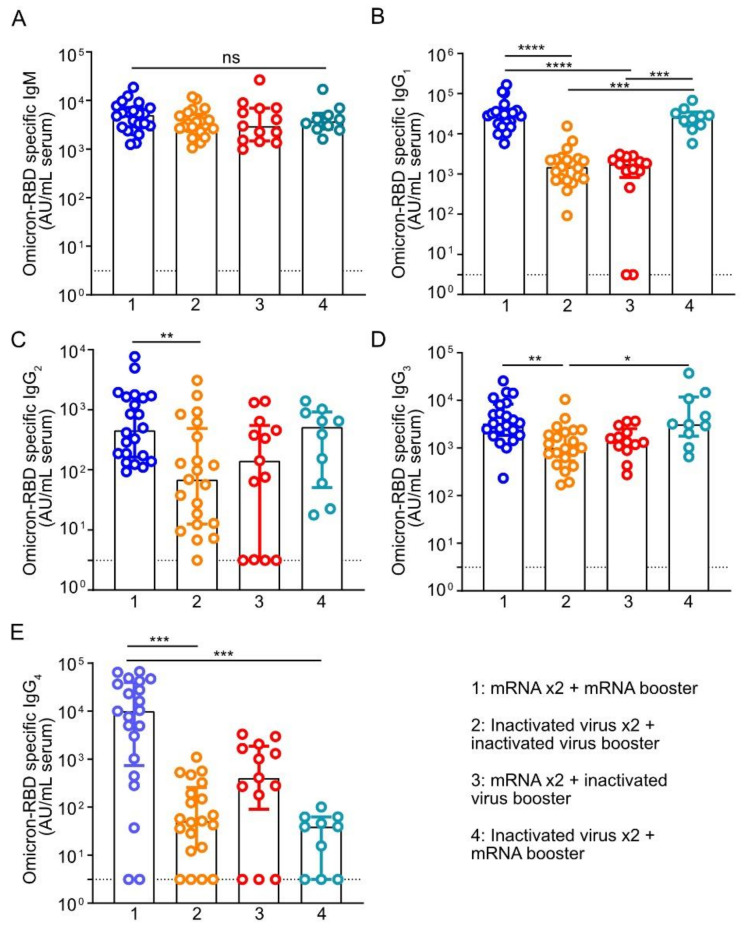
Antibody levels in individuals receiving third dose mRNA or inactivated virus vaccine booster. Comparison of (**A**) IgM or (**B**) IgG1 (**C**) IgG2 (**D**) IgG3 and (**E**) IgG4 against RBD of Omicron variant of SARS-CoV-2 spike protein in plasma samples obtained 28 ± 5 days post-immunization from individuals receiving (1) three doses of mRNA vaccines; (2) three doses of inactivated virus vaccines; (3) two doses of mRNA vaccines followed by a booster shot with inactivated virus; and (4) two doses of inactivated virus vaccines followed by a booster shot with mRNA vaccine. Each symbol represents a unique individual. Bars represent median antibody levels, and error bars show interquartile range (IQR). AU = arbitrary units. Statistical tests: Kruskal–Wallis test whereby * *p* < 0.05, ** *p* < 0.01, *** *p* < 0.001, **** *p* < 0.0001 and ns = not significant. The dotted line indicates a lower limit of detection of the assay (AU 3.125).

We first quantified antibodies against the Omicron RBD in the plasma using ELISA. We found that levels of IgM against Omicron RBD were similar regardless of the vaccine regimen, while notable differences exist for IgG antibodies against Omicron RBD (Figure 1A–E). Firstly, three homologous doses of mRNA vaccines resulted in the highest median levels of all IgG subclasses compared with other vaccination regimens (Figure 1B–E). In particular, donors who received three doses of mRNA vaccines (Group 1) had significantly higher median levels of IgG1 (*p* < 0.0001), IgG2 (*p* = 0.0087), IgG3 (*p* = 0.0014), and IgG4 (*p* = 0.0002) than individuals receiving three doses of inactivated virus vaccines (Group 2) (Figure 1B–E). IgG1 is the most abundant antibody in the human plasma, comprising approximately 60–70% of total IgG levels and playing an essential role in protection against bacterial and viral infection.

More importantly, we found that individuals who received homologous or heterologous mRNA booster shots had about 17–19-fold higher median Omicron RBD-reactive IgG1 (Group 1: median 28,735 [Interquartile range (IQR) 17,181–36,627] and Group 4: median 25,650 [IQR 15,776–34,747]) compared to individuals receiving homologous or heterologous inactivated virus booster shots (Group 2: median 1512 [IQR 727–2546] and Group 3: median 1786 [IQR 822–2475]) after two doses of priming mRNA vaccines or inactivated virus vaccine (Figure 1B). Similarly, mRNA booster regimens also resulted in at least 3-fold higher median Omicron RBD-specific IgG3 compared to boosters with the inactivated virus (Figure 1D). Individuals in Group 1 (median: 3305 [IQR 1840–8283]; *p* = 0.0014) and Group 4 (median: 3155 [IQR 1768–11,782]; *p* = 0.0227) had significantly higher levels of IgG3 compared to individuals in Group 2 (median: 1014 [IQR 536.3–2256]) (Figure 1D). Notably, only levels of RBD-reactive IgG1 and IgG3 but not IgG2 and IgG4 were significantly increased upon booster with mRNA vaccine regardless of whether an individual had received priming doses of mRNA (Group 1) or inactivated virus vaccines (Group 4).

### 3.3. Homologous or Heterologous mRNA Booster Dose but Not Booster Dose with Inactivated Virus-Induced Neutralizing Antibodies against the Omicron RBD

**Figure 2:**
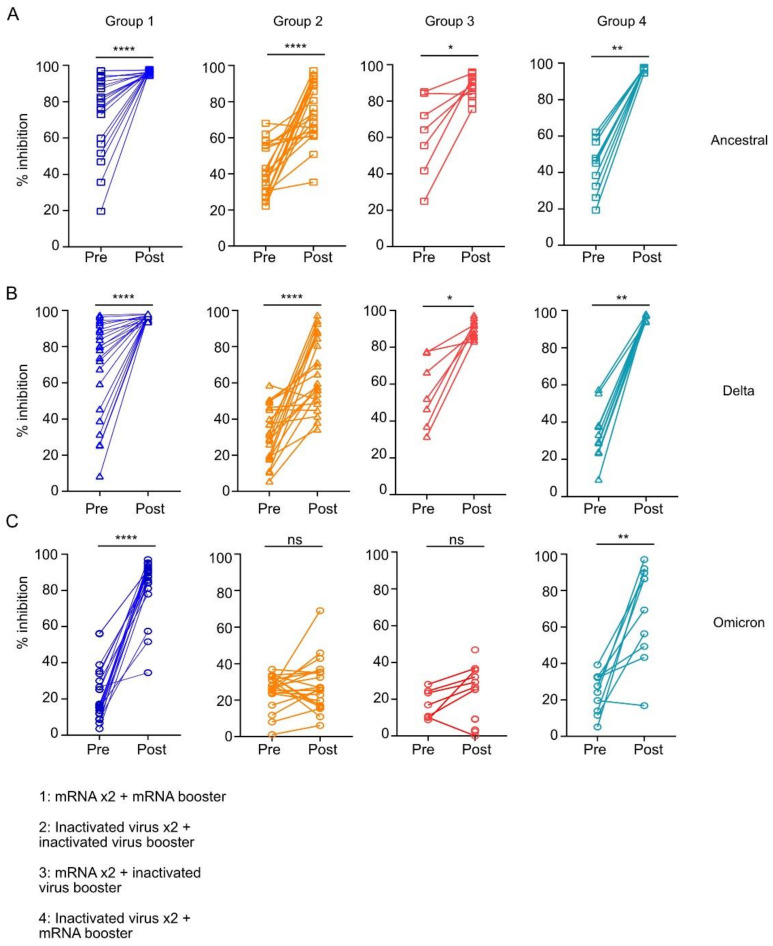
Neutralizing activity of antibodies against RBD of the original strain, Delta and Omicron variant of SARS-CoV-2 following mRNA or inactivated virus vaccine booster in individuals previously immunized with two doses of mRNA or inactivated virus vaccine. Comparison of percentage inhibition of antibody binding to RBD of spike protein by (**A**) original strain (**B**) Delta variant or (**C**) Omicron variant, in plasma samples obtained 1–7 days before booster vaccination and 28 ± 5 days post booster vaccination from individuals receiving (1) three doses of mRNA vaccines; (2) three doses of inactivated virus vaccines; (3) two doses of mRNA vaccines followed by a booster shot with an inactivated virus vaccine; and (4) two doses of inactivated virus vaccines followed by a booster shot with mRNA vaccine. Two symbols connected by a line represent repeated samples from a unique individual pre and post-booster vaccination. Only 7 out of 13 individuals provided pre-booster blood samples in Group 3. AU = Arbitrary Units. Statistical tests: Wilcoxon matched-pairs signed-rank tests whereby * *p* < 0.05, ** *p* < 0.01, **** *p* < 0.0001 and ns = not significant.

To understand if these antibody levels correlated with neutralizing activities against the VOC, we evaluated neutralizing capabilities of the antibodies induced by the various booster regimens (Figure 2A–C) using the cPass assay [14]. Briefly, plasma antibodies were incubated with either RBD from Wuhan, Delta or Omicron strain of SARS-CoV2 before assessing for their ability to bind to the respective RBD coated on the immunosorbent plates. Evaluation of total neutralizing antibodies was assessed by % inhibition of antibody binding to RBD on the plate by prior incubation with the RBD from either Wuhan, Delta, or Omicron strains. Using the cPass assay, we measured the neutralizing capabilities of antibodies against the ancestral strain of SARS-CoV2 (Wuhan-Hu-1) or VOCs (Delta B.1.617.2 or Omicron B.1.1.529) in individuals before receiving the booster shot and 28 ± 5 days after receiving their booster dose of either mRNA or inactivated virus vaccine. Irrespective of initial regimens, all booster shots led to an overall increase in antibody neutralizing capability against Wuhan-Hu-1 and the Delta variants of SARS-CoV-2 (Figure 2A,B). Notably, the mRNA booster increased the median percentage of neutralization against the Wuhan-Hu-1 and Delta variants up to more than 90% in individuals who previously received two doses of mRNA (Group 1) or inactivated virus (Group 4) vaccines (Figure 2A,B). Similarly, a booster shot with the inactivated virus vaccine could also increase the median percentage of neutralization against the Wuhan-Hu-1 and Delta variants to above 80% in individuals who previously received two doses of mRNA vaccines (Group 3). However, the effectiveness of the third dose of CoronaVac or BBIBP-CorV in individuals receiving two previous doses of CoronaVac (Group 2) was variable and modest, reaching a median percentage of neutralization of 76.30% (IQR 62.90–91.65%) and 64.90% (IQR 49.6–88.05%) for the Wuhan-Hu-1 strain and Delta variant, respectively, 28 ± 5 days post booster (Figure 2A, 2B).

Consistent with data demonstrated by other groups [6,9,11,15,16], we also found that two doses of either mRNA or viral inactivated vaccines had poor levels of neutralizing antibodies against the Omicron variant when assessed between 3–6 months after the second dose of vaccine (Group 1, median 16.00% [IQR 11.97–30.98%]; Group 2, median 26.50% [IQR 23.55–32.05%]; Group 3, median 16.90% [IQR 10.06–24.50%]; and Group 4, median 25.84% [IQR 13.30–32.63%]).

It is critical to note that only the mRNA vaccine booster was able to effectively increase the median levels of neutralizing capabilities against the Omicron variant (Group 1, median 88.80% [IQR 83.28–92.45%], *p* < 0.0001; Group 4, median 77.85% [IQR 47.80–90.28%], *p* = 0.0039) by 5.5-fold and 3-fold, respectively, post booster (Figure 2C). In contrast, an inactivated virus booster shot was unable to significantly increase the median levels of neutralizing capabilities against the Omicron variant regardless of whether the individuals received two priming doses of mRNA vaccines (Group 3, median 25.40% [IQR 6.200–33.95%]) or two priming doses of inactivated virus vaccines (Group 2, median 26.40% [IQR 16.80–35.30%]) (Figure 2C).

### 3.4. Frequencies of Omicron RBD-Binding B Cells Were Higher in Individuals Receiving Homologous or Heterologous mRNA Booster Doses Regardless of the Priming Vaccines

**Figure 3:**
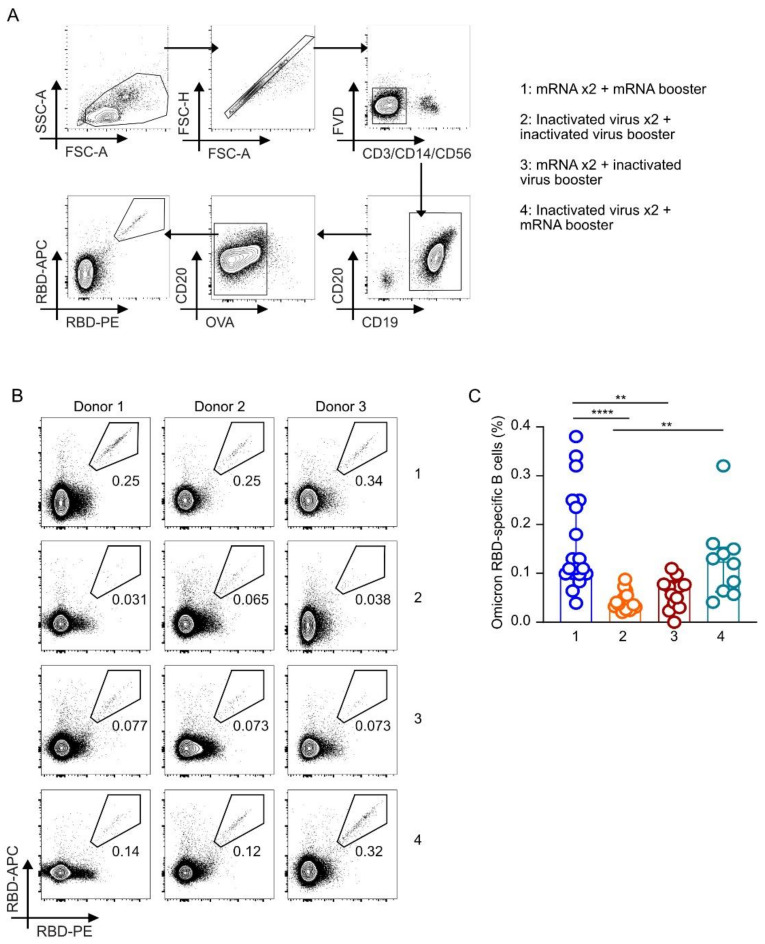
SARS-CoV-2 Omicron RBD-binding B cells in individuals receiving various homologous and heterologous booster vaccines. (**A**) Gating strategy used for RBD-binding B-cell detection. Lymphocytes were first gated with FSC-A versus SSC-A. Single cells were then identified with FSC-A versus FSC-H. We then selected live Dump^-^ cells with FVD-BV510 versus Dump (anti-CD3, -CD14, and -CD56)- BV650. Total CD19^+^CD20^+^ B cells were subsequently gated from live Dump^-^ cells with CD19-BV421 versus CD20-BUV395. We then selected OVA^-^ B cells using OVA-PE-Cy7 versus CD20-BUV395. SARS-CoV-2 Omicron RBD double-positive cells were further identified from OVA^-^ B cells using RBD-APC versus RBD-PE. (**B**) Representative flow plots showing dual APC-RBD- and PE-RBD-binding B cells for twelve unique individuals from four different vaccination groups. Numbers adjacent to the gate indicate the percentage of cells within the gate over the total B cells analyzed. (**C**) Percentages of SARS-CoV-2 Omicron RBD-binding B cells. The percentages of Omicron RBD-binding B cells in individuals from four different booster vaccination groups were summarized based on the flow cytometry analysis. Bars represent median frequencies of RBD-binding B cells, and error bars show interquartile range (IQR). Statistical tests: Kruskal–Wallis test whereby ** *p* < 0.01 and **** *p* < 0.0001.

Lastly, we compared the frequencies of Omicron RBD-binding B cells in individuals receiving different booster vaccines based on the gating strategy described in Figure 3A. We observed that corroborating our antibody data, the frequencies of B cells that can bind to the Omicron-RBD were present at significantly higher levels in groups that received three homologous mRNA vaccines as a booster shot in Group 1 (median 0.11% [IQR 0.099–0.25%]) compared to individuals receiving three homologous inactivated virus vaccine booster shots in Group 2 (median 0.038% [IQR 0.032–0.052%], *p* < 0.0001) or a heterologous inactivated virus vaccine booster after two priming doses of mRNA vaccines in Group 3, (median 0.073% [IQR 0.035–0.078%], *p* < 0.0037). Similarly, individuals who received a heterologous booster of mRNA vaccine after two priming doses of inactivated virus vaccine in Group 4 (median 0.13% [IQR 0.062–0.15%], *p* = 0.0011) also had higher frequencies of the Omicron RBD-binding B cells than individuals who received three homologous doses of inactivated virus vaccine in Group 2. Notably, there is also no statistical difference between individuals who received homologous or heterologous mRNA vaccine booster shots regardless of whether they received two doses of priming mRNA or inactivated virus vaccines (Figure 3B,C).

Collectively, our data showed that although booster vaccination with the inactivated virus can help increase neutralizing antibodies against ancestral or Delta strains of SARS-CoV2, booster vaccination with mRNA vaccine is vital in inducing protective B-cell responses against the Omicron variant.

## 4. Discussion

Several recent studies have demonstrated a considerable reduction in the neutralization abilities of vaccine-elicited antibodies against the Omicron variant compared to ancestral or Delta strains [8,15]. In addition, the Omicron variant showed considerable immune escape from convalescent individuals [9]. However, booster regimens with mRNA vaccines, BNT1626b and mRNA-1273, have been shown to improve the neutralization of the Omicron variant by antibodies elicited in individuals who had previously received two doses of mRNA vaccines or been infected with SARS-CoV2 [6,9,11,17]. These results suggest the necessity of a booster vaccine for fully vaccinated or convalescent individuals to reduce the risk of symptomatic breakthrough infections by the Omicron variant.

To the best of our knowledge, there has been no prior study that compares different vaccination regimens involving homologous mRNA or inactivated virus vaccines and heterologous mRNA plus inactivated virus vaccines in a single population. In this study, we characterized the humoral immune responses in terms of antigen-specific antibody production, neutralizing capability against the Omicron variant, and the frequency of the Omicron RBD-binding B cells in individuals who received homologous or heterologous mRNA and inactivated virus vaccines.

In agreement with previous studies, we found that plasma from individuals who received homologous mRNA booster vaccination contains antibodies with a greater ability to neutralize the Omicron variant [9,11,17]. More interestingly, we demonstrated that a significant increase in median neutralizing capability (3.01-fold, up to 77.85% of neutralization) against Omicron RBD could also be achieved in individuals who received an mRNA booster after two doses of inactivated virus vaccines. In contrast, individuals receiving inactivated virus vaccine booster following two doses of priming inactivated virus vaccines did not have a substantial increase in neutralizing capability against the Omicron variant despite significant increases in that against the ancestral strain or Delta variant. This finding is similar to a study by Perez-Then et al. (2022), who did not detect neutralizing antibodies against the Omicron variant in individuals who received two doses of Coronavac but found a 1.4-fold increase in neutralization activity against Omicron in individuals who received two doses of CoronaVac followed by a booster with BNT162b2 compared to individuals who received two doses of mRNA vaccine [15]. This is also corroborated by another study by Zuo et al. (2022), who observed increases in antibodies that can bind the RBD of VOCs, including Delta (8-fold) and Omicron (14-fold), in 13 individuals who received an mRNA booster after a two-dose regimen of inactivated virus vaccine [16]. In our current study, we found that the median Omicron RBD-specific IgG1 levels robustly increased by approximately 17–19-fold in individuals approximately one month after receiving an mRNA booster following two doses of mRNA or inactivated virus priming vaccination. As IgG1 makes up about 60–70% of all IgG in the blood and plays a role in protection against infection by pathogens, changes in levels of this dominant antibody isotype in the blood are likely to correlate with the corresponding increase in neutralizing capabilities against the Omicron variant in these two groups of vaccinated individuals.

One surprising finding to us was a similar lack of neutralizing abilities against the Omicron variant in individuals who had been primed with two doses of mRNA vaccines but boosted with inactivated virus vaccine. Corroborating this finding, the frequencies of Omicron RBD-binding B cells in the individuals reflect this pattern. These data prompted our speculation that, unlike the mRNA vaccines, inactivated virus vaccines could be less potent in inducing robust antibody recall responses and further affinity maturation after an individual had received the initial two doses of vaccines, including mRNA vaccines, which target the ancestral spike protein of the SARS-CoV-2. These hypotheses will have to be further tested using B cell receptor sequencing and clonal analyses to understand changes in the B-cell repertoire induced by the different booster vaccination types.

Since lower neutralizing antibody titers have been shown to correlate with increased risk of symptomatic COVID-19, individuals who underwent two-dose vaccination regimens and have yet to receive an mRNA vaccine booster may have limited neutralizing activity against the Omicron variant and be at a higher risk of breakthrough infections with higher disease burden [18]. Collectively, our data suggest that, in order to prevent breakthrough infections by the Omicron variant, countries that predominantly use the inactivated virus vaccines could consider alternative vaccination strategies to boost immune responses in vaccinated individuals. In addition, to ensure that individuals with adverse reactions, such as anaphylactic responses to mRNA vaccines, can still be protected from emerging VOC, the efficacy of heterologous vaccine boosters with alternatives such as protein-based or adenoviral vector-based vaccines should be studied since our data suggest that inactivated virus booster vaccination is ineffective in inducing neutralizing antibodies against the Omicron variant in individuals who had received two priming doses of mRNA vaccines. Lastly, a recent study demonstrated high preservation of T cells that recognize epitopes on Omicron RBD, suggesting that protection of T-cell immunity can play an important role against this VOC [19].

We acknowledge that our current study is limited to humoral responses, and it is important to understand whether anti-viral immunity mediated by T cells and other immune cells is different in the groups receiving homologous and heterologous priming and booster vaccination regimens. We acknowledge that this study was conducted with a relatively small number of individuals in each group, in particular for Groups 3 and 4, which consisted of the individuals who opted for heterologous prime and booster regimens (Group 1: 22; Group 2: 21; Group 3: 13; Group 4: 10). Future studies with larger sample sizes that include different geographical groups are necessary to investigate further whether the results obtained in the current study can be extrapolated. Additionally, our donors consisted of healthy adults with an age range of 32–60. Hence, it is important to know whether the observations remain the same in bigger study groups or among individuals from different age groups. It is also critical to understand whether the durability of humoral responses against the Omicron variant differs in groups receiving different priming and booster vaccination regimens.

## 5. Conclusions

From our observations, we robustly show that individuals who received mRNA booster vaccination, regardless of the type of vaccines (mRNA or inactivated virus) they received for priming dose, were able to produce higher levels of Omicron RBD-specific antibodies with the ability to neutralize the Omicron variant of SARS-CoV2. This likely confers higher protection against symptomatic infection caused by the Omicron variant in these individuals. On the contrary, individuals receiving inactivated virus booster vaccination were unable to produce robust levels of antibodies capable of neutralizing the Omicron variant. This may also translate to lower protection against symptomatic breakthrough infections by the Omicron variant. As such, alternative vaccination strategies need to be explored for individuals who received inactivated virus vaccine boosters and those who were unable to receive booster vaccination of the mRNA type to maintain humoral immunity against SARS-CoV2 VOC. Further work is necessary to understand the longevity as well as the cellular immune responses of the different priming–booster vaccination regimens against the VOC.

## Figures and Tables

**Table 1:** Characteristics of study participants.

Vaccine Regimen	3 Doses of mRNA (*n* = 22)	3 Doses of Inactivated Virus (*n* = 21)	2 doses of mRNA + Inactivated Virus Booster (*n* = 13)	2 Doses of Inactivated Virus + mRNA Booster (*n* = 10)
Age, years	38 (32–51)	41 (37–48)	52 (45–60)	40 (28–49)
Sex				
Male	11 (50.0)	7 (33.3)	6 (46.2)	5 (50.0)
Female	11 (50.0)	14 (66.7)	7 (53.8)	5 (50.0)
Ethnicity				
Chinese	20 (90.9)	21 (100)	13 (100)	10 (100)
Indian	1 (4.5)	0	0	0
Others	1 (4.5)	0	0	0
Time from the second dose to booster, months	5.5 (5.0–6.3)	3 (2.5–3.0)	6 (6.0–7.0)	3 (3.0–3.5)

Data are expressed as median (IQR) or *n* (%).

## Data Availability

Data generated from this study are available upon reasonable request from the corresponding author.
